# Simulation Training in Video-Assisted and Robotic-Assisted Cardiac Surgery: A Narrative Review

**DOI:** 10.3390/jcdd13050180

**Published:** 2026-04-26

**Authors:** Fatemeh H. Nameghi, Jason M. Ali

**Affiliations:** 1 Department of Cardiothoracic Surgery, Royal Papworth Hospital, Cambridge CB2 0AY, UK; fatemeh.nameghi@nhs.net; 2 School of Clinical Medicine, University of Cambridge, Cambridge CB2 0SP, UK

**Keywords:** minimal access cardiac surgery, simulation, training

## Abstract

Minimal access cardiac surgery (MACS) can mitigate the increasing risk profile of cardiac surgery patients and is associated with improved postoperative outcomes. One of the ways to manage the steep learning curve of MACS is the use of surgical simulation training. We conducted a narrative review to identify the relevant literature discussing MACS simulation training. We identified 20 studies using our search strategy. Various platforms were represented: high-fidelity (*n* = 8), low-fidelity (*n* = 6), and animal studies (*n* = 6). Virtual reality (VR) appeared in two wet-lab studies as an adjunct. The surgical approach was video-assisted thoracoscopic surgery (VATS) in 11 and robotic-assisted thoracoscopic surgery (RATS) in nine. The most simulated procedure was minimal access mitral valve (MV) repair (*n* = 16). Most studies (*n* = 16) evaluated the impact of simulation training on the surgical skill of participants with varying baseline MACS experience. A small proportion of included studies (*n* = 4) carried out only fidelity testing. While some standardised assessment tools were used, there was considerable variation in how surgical skill and fidelity were assessed. There are an increasing number of publications on MACS simulation training, with equal focus on bench and animal models. MV procedures were the most simulated, suggesting a drive towards increasing the scope of minimal access MV training.

## 1. Introduction

Despite the increasing risk profile of patients, outcomes following cardiac surgery continue to improve worldwide. This is related to improvements in preoperative optimisation, surgical practice, and perioperative management. As has been seen for all surgical specialties, there has been a move towards more minimal access approaches to undertaking cardiac surgery—with both video-assisted (VATS) and robotic (RATS) techniques being increasingly adopted as an alternative to the traditional median sternotomy. The reported advantages of these techniques include reduced surgical trauma and postoperative pain, lower infection risk, superior cosmesis, and possibly, reduced length of stay [1]. These factors are contributing to increasing patient and referring physician demand for minimal access cardiac surgery (MACS) [2].

As has been seen in other surgical specialties transitioning to minimal access techniques, there is a steep learning curve, and new skills must be learnt by surgeons used to operating open. There are different skills required by surgeons performing VATS procedures. This is due to having a restricted two-dimensional field of view, reduced tactile sensation and feedback, and requiring familiarity with long-shafted, straight instruments. For RATS, there are again additional skills required.

For various reasons, including reduced resident doctor working hours, simulation has become an integral part of surgical training, allowing surgeons and surgical trainees to develop surgical skills outside of the operating theatre. There are many advantages of this approach to surgical training, including the opportunity to acquire surgical dexterity in safe environments, minimising potential risk to patients. In addition, there is opportunity to make objective assessment and gain individualised feedback of simulated surgical skills.

Over recent years simulators of various fidelity have been developed in open cardiac surgery, from simple coronary anastomosis simulators using rubber tubes, through to live operating on porcine models [3,4]. Fidelity here refers to the degree to which the simulator and surrounding environment resemble operating room conditions, and it can be broadly split into high-fidelity simulation (HFS) and low-fidelity simulation (LFS). HFS may be perceived by some to be superior to LFS due to its closer resemblance to reality in terms of anatomical accuracy, as well as how real it feels to use, i.e., psychological fidelity.

With the increasing trend towards minimal access techniques, a demand has developed for MACS simulators. In this review, we aim to describe the current scope of simulation training in robotic-assisted and video-assisted MACS through a discussion of the range of simulation models and assessment tools reported in the literature, also considering trainee perspectives.

## 2. Materials and Methods

A narrative review was conducted. We searched PubMed, Embase, and Web of Science using MESH terms and keywords. Full search criteria is provided in Supplementary Tables S1–S3. Studied discussing VATS or RATS simulation in adult cardiac surgery for training purposes were included. Full exclusion criteria are provided in the adapted PRISMA diagram in Supplementary Figure S1. Articles were limited to those in the English language and published between 1995 and 2026. We identified 20 studies using the search strategy. Table 1 and Table 2 summarise the findings of studies simulating VATS and RATS procedures, respectively. The details of included models, including materials used, assessment tools, and simulation cost, is provided in this section to supplement the methodology.

### 2.1. Simulation Materials

Many HFSs are 3D-printed from CT-derived anonymised patient data. The use of 3D models within MACS allows a tactile perception of complex pathologies which is not possible through computerised 3D reconstructions, with higher anatomical accuracy than is permitted by LFS [25]. The use of CT-derived data also permits simulation of patient-specific anatomy, which can be valuable in the preoperative planning of complex procedures. While the one-off cost of printers can be quite high, printing materials can be relatively cheap. Polyvinyl alcohol (PVA) is often used with injection moulding due to being water-resistant and readily available. Unlike silicone, it does not require specialist printing equipment. However, silicone can offer more realistic tensile strength and elasticity than PVA where cardiac structures are concerned [26]. In the included studies, the 3D printing approach was used primarily for simulating mitral valve (MV) procedures [7,9,10,21,22,24].

In contrast, the LFS models evaluated in Table 1 and Table 2 used commonly found household items such as baby bottles and associated feeding teats [13], as well as Lego^®^ (Billund, Denmark) classic blocks and Lego^®^ Technic™ parts (The LEGO Group), and kitchen sponges [13]. Verberkmoes and Verberkmoes-Broeders [5] evaluated their baby bottle model for video-assisted MV and tricuspid valve surgery, which cost $9 per simulator. The baby bottle acted as a holder for the MV structures, while the feeding teat represented the annulus and subvalvular apparatuses; a dental dam (commonly used in endodontic surgery) was used to simulate the leaflets of the MV. The simulator’s fidelity was validated by comparing the suture tension and tactile response to those of the human MV, which were found to be adequately similar.

### 2.2. Assessment Tools

Simulation in MACS needs to be paired with fidelity testing and standardised tools to assess surgical performance. Table 3 summarises commonly used tools within the included literature to assess technical skill and perceived competence, as well as the structural fidelity of simulators.

The GEARS scale was created by Goh et al. [27], who adapted the Global Assessment of Laparoscopic Skills (GOALS) scale [28] for assessment of elements of robotic surgery. GEARS has six elements, including depth perception, bimanual skill, efficiency, force control, autonomy, and robot control. All of these elements, except depth perception, are reported to differentiate between individuals based on their level of expertise (e.g., consultants and trainees) [29]. The 3D vision afforded by modern robotic systems may compensate for the less experienced depth perception of trainees, therefore leading to similar scores between trainees and consultants. This led to the adaptation of GEARS into the modified GEARS (mGEARS), which excludes depth perception as a parameter. The included studies used both GEARS and mGEARS in conjunction with animal and virtual reality (VR) models [16,17,18], suggesting the adaptability of both scales to various simulation platforms.

### 2.3. Simulation Cost

The included studies highlight a lack of economic transparency in MACS simulation literature. While the approximate cost per-cadaver during wet-lab sessions is a well-known premium, the same transparency is not afforded to bench models or VR interfaces. The cost of well-known minimal access cardiac surgery platforms such as the Simurghy trainer, for example, is not available to the public. From the included literature, seven explicitly stated the cost of their simulation platforms (Table 4).

## 3. Results and Synthesis

### 3.1. Results Summary

**Figure 1:**
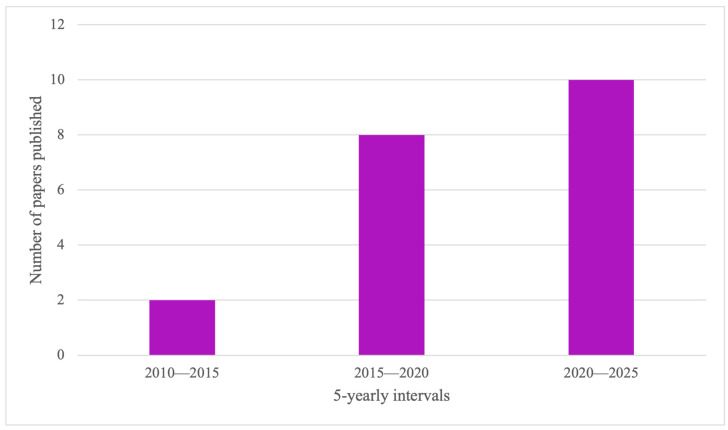
Graph depicting the number of studies published in minimal access simulation training (VATS and RATS) in adult cardiac surgery in the recent years.

We identified 20 studies using the search strategy. Table 1 and Table 2 summarise the findings of studies simulating VATS and RATS procedures, respectively. Various simulation platforms were represented. HFS was used in eight studies, while LFS and animal models (porcine and calf) both appeared in six studies each. VR was used as an adjunct to wet labs in two studies. There were eleven studies simulating VATS procedures, and nine simulating RATS. Valvular procedures were the most simulated, with 16 studies simulating some aspect of MV replacement/repair. The number of papers published on VATS and RATS simulation training in adult cardiac surgery appears to be rising in recent years as per Figure 1.

### 3.2. Surgical Procedure Frequency

The representation of MV procedures in the literature may arise from a combination of procedural difficulty (MV procedures may often involve intricate repair rather than total replacement [30]) and the higher volume of minimally invasive MV surgery (MIMVS) performed compared to other valves including aortic.

The technicalities of minimal access MV repair include, but are not limited to, maintaining a mini-thoracotomy incision of less than 5 cm, as well as the technique of aortic occlusion within limited space. The success of surgical outcomes of minimal access MV procedures is reported to directly correlate with hospital volume of minimal access MV cases [31]. Cases with complex anatomical pathologies are often deemed high risk and referred to experienced centres.

This creates a vacuum of opportunities for trainees at lower-experience centres to increase their exposure to the true breadth of minimal access MV surgery. The frequency of simulated minimal access MV operations in the literature may be reflective of the high demand for out-of-theatre training opportunities.

The British and Irish Society for Minimally Invasive Cardiac Surgery (BISMICS) consensus statement on minimal access MV training in the UK states that compared to sternotomy, patients undergoing minimal access MV surgery experience lower wound sepsis and postoperative bleeding. This is paired with higher patient satisfaction related to a quicker return to activities of daily living due to a shorter postoperative recovery period [32]. BISMICS recommend team-based simulation and wet-lab sessions to increase trainee familiarity with long-shafted instruments.

This can increase suture accuracy and, therefore, decrease the time taken for procedures such as ring annuloplasty and leaflet plasty [7]. Several studies in the literature showed that both time and accuracy in performing MV tasks could be significantly improved with both LFS and HFS [7,8,18,20].

Robotic internal mammary artery (IMA) harvest was also commonly represented (17–20). The prevalence of robotic IMA harvest in the literature may reflect increased training in minimal access techniques for long-established procedures such as CABG.

### 3.3. Levels of Simulators

Simulation can be categorised based on fidelity and biological format/platform [33]. The fidelity of surgical simulation (low or high) is often correlated with its closeness to reality. LFSs may be classed as those allowing simulation of only simple skills, while HFSs allow a more realistic simulation of an operation from start to finish. This approach to fidelity considers only physical fidelity, however. Fidelity, as a whole, also concerns subjective operator-dependant measures such as how realistic a simulator feels to use: psychological fidelity [34].

While none of the included studies directly compared HFS and LFS, it is generally agreed that an increasing level of fidelity is associated with increasing cost but not necessarily with superior training value [35,36]. The ideal simulator should strike the perfect balance between keeping costs low and training value high [34].

Simulation may utilise biological or non-biological platforms. Biological platforms include animal models and human cadavers, while non-biological formats include bench models and VR simulators. While human cadaveric training is considered the gold standard simulation technique for surgical training [37], facility hire and cost per cadaver limit its availability. These barriers, along with ethical considerations, make it extremely rare for this training to be at the disposal of cardiac surgery trainees worldwide. This may, in the near future, encourage a shift away from cadaveric training as the gold standard and towards integrated surgical training programs which more readily utilise various types of simulation.

Of the animal models appearing in the literature, models of porcine origin were the most common [16,17,18,19,23], while one study utilised a foetal bovine calf model [20]. The wider literature does not explicitly compare bovine and porcine models for simulation. Most comparisons made between bovine and porcine hearts relate to their performance as bioprosthetic valves [38,39] which is outside the remit of this review.

Animal models significantly reduced the sequential operating times of robotic IMA harvest in a calf model in a study carried out by Ashraf et al. [20]. When comparing animal models to other simulation platforms, in one randomised controlled trial (RCT) comparing VR and porcine models for IMA robotic harvesting and MV annuloplasty, trainees were randomised to one of the following simulation environments: wet lab (using a porcine model), dry lab, or VR [17].

The wet-lab group showed the greatest improvement in time-based scoring (TBS) assessment. Although the VR group did meet the levels of proficiency set by the study experts for TBS and GEARS, this was not as high as the wet-lab group. In another RCT by the same group, candidates assigned to practice with VR before assessment of IMA harvest and MV annuloplasty on a porcine model performed significantly better than the control group who did not undergo VR training beforehand [16].

VR as an adjunctive platform may ensure that trainees can make the most of expendable wet-lab materials by familiarising themselves with the procedure beforehand. VR allows for repeated practice of focused skills without using up consumables (as with simulators) or altering the surgical anatomy of the simulation field (as in wet labs), allowing all participating trainees to have an equal learning experience.

In a multi-centre evaluation of U.K.-based minimal access cardiac surgery courses by Moorjani et al. [40], the courses with access to the live operating simulation models rated highest in scoring for content and materials as well as for organisation and facilities.

High-fidelity bench models were often used in the literature for minimal access MV surgery procedures [6,8,9,10,11]. As discussed in the previous section, the prevalence of such models for minimal access MV surgery may arise from the lack of trainee access to MV cases [41].

HFS improved the time taken and accuracy of annular sutures placed with long-shafted instruments in two proof-of-concept studies [8,10], and the speed of knot tying in another [15]. In a study by Nia et al. [8], consultants and residents improved their time and anatomic accuracy in placing anterior and posterior annular sutures using long-shafted instruments in a model thoracic torso for MIMVS. In a study using a 3D-printed telesimulator including a printed valvular apparatus and left ventricle, Cheheili Sobbi et al. [10], demonstrated that 100% of their MACS experts and trainees could place an anatomically correct suture post-simulation training compared to only 48% before, with a significant improvement in the time taken to place sutures as well.

In another study by Sobbi et al. [9], HFS improved the self-reported confidence levels of both trainees and consultants in suture placement, valve implantation, and suture knotting using a knot pusher. A group of 25 medical students in Wang et al.’s [11] study also experienced significant improvement in neochorda implantation and annuloplasty sutures and demonstrated shorter duration of knot tying after HFS training, suggesting the educational value of simulation across all training levels.

### 3.4. Trainee Perception

In a country-wide survey of surgeons and trainees at 12 institutions in Canada, a questionnaire was aimed at unveiling trainee perceptions and confidence levels when it came to simulation training [42]. The survey saw that the majority of participants indicated a desire for expanded simulation training at their centres [42]. There was a prevalent belief that being able to practice the required skills before operating would improve theatre confidence in residents. Similarly, seniors indicated they would feel more comfortable with allowing residents to operate on patients if they knew residents had undergone simulation training beforehand. This suggests that simulation training may act as a gateway towards theatre opportunities for residents, not only through improvements in their own confidence but also through that of their seniors’ in them. Using an LFS for MV and aortic valve (AV) replacement in a study by En Yean et al. [12] and a calf model for IMA harvest in a study by Ashraf et al. [20], participants experienced significantly increased scores on SSES and PCS scales following simulator training.

## 4. Discussion

**Figure 2:**
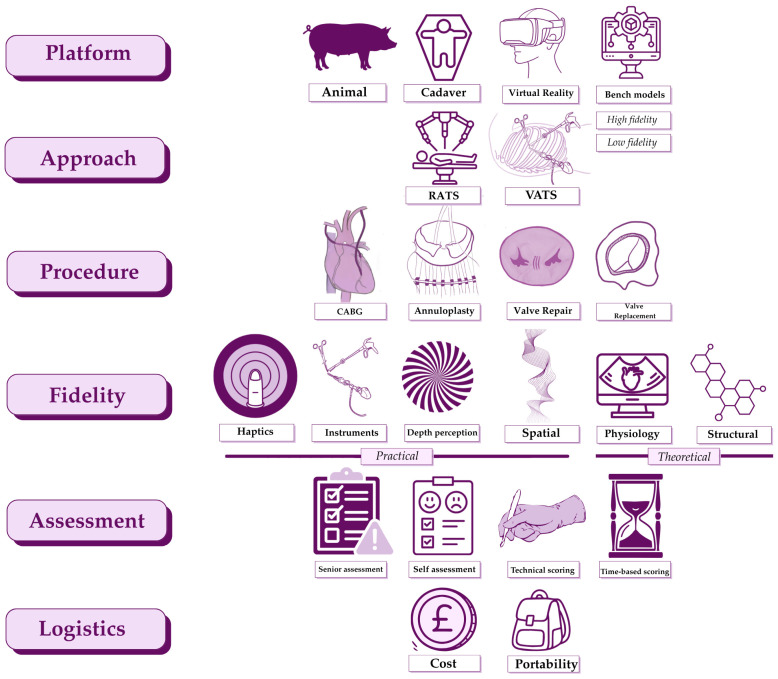
Concept map for simulation training in minimal access adult cardiac surgery. Our review finds simulation training in minimal access cardiac surgery training to be defined by six major components. These are: simulation platform i.e., animal or cadaveric; surgical approach i.e., robotic or video-assisted; type of procedure simulated; fidelity of simulation; assessment tools used to evaluate simulation users; as well as the logistics of both local and wide-spread implementation of the simulation in question as a training tool. The fidelity of the platform is a multifactorial property which is defined by haptics, the usability of long-shafted instruments within the simulation field, depth perception, the realism of spatial limitations to those in theatre, as well as the structural and physiological resemblance of the simulator to real-life. Figure created with Canva Visual Suite 2.0 and Procreate for iPad version 5.4.8 (Savage Interactive Pty Ltd., Tasmania, Auatralia). VATS, video-assisted thoracoscopic surgery; RATS, robotic-assisted thoracoscopic surgery; CABG, coronary artery bypass grafting.

Simulation training in MACS provides budding surgeons with a safe environment to practice requisite skills. The ideal simulation platform mirrors the real-life operating environment while allowing repeated practice of both focused skills and whole procedures under expert guidance. The included literature evaluates VATS and RATS simulation platforms for valvular and coronary procedures [43,44]. The methods used include assessment of surgical performance and isolated fidelity testing. The various components of simulation training in VATS and RATS adult cardiac surgery are summarised in Figure 2.

The main limitation of the included papers is the heterogeneity of assessment tools. used. For example, both GEARS and mGEARS do not include any time-based parameters. Cross-clamp and bypass times are important in contributing to cardiac surgery outcomes, and therefore, time does matter in cardiac surgery. Atroshchenko et al. [18] carried out a multicentre trial using porcine simulation where both trainees and experienced participants carried out left atrial (LA) closure, IMA harvest, and MV annular suturing. They found that mGEARS and TBS assessments could both discriminate between different levels of competence in LA closure and MV annular suturing but not IMA harvest.

This raises an important point: technical scoring systems may not bear the same utility in evaluating all surgical tasks. On two separate occasions, Valdis et al. [16,17] used GEARS to evaluate the impact of simulation raining on IMA dissection skill in trainees and found a significant improvement in the GEARS scores of participants who underwent adjunctive VR and wet-lab training prior to assessment.

In contrast, Atroshchenko et al. [18] suggest that there was insufficient validity evidence to support the assessment of IMA harvest using GEARS in their study. This cannot be attributed solely to GEARS, and the simulation platform used may have a part to play in the contrasting results of Valdis et al. [16,17] and Atroshchenko et al. [18].

One solution could be the pre-validation of scoring systems for the specific task being assessed. This could be achieved by comparing the baseline scores of consultants and trainees to validate the efficacy of the score in discriminating competence levels in that task.

Some studies paired technical scoring scales, such as the Objective Structured Assessment of Technical Skills (OSAT), with psychological scales such as the perceived competence scale (PCS) or surgical self-efficacy scale (SEES) [12]. While self-evaluation may not be as reliable as senior evaluation, such hybrid assessment tools allow for the consideration of trainee perspectives. This may shed light on the perceived educational value of these platforms by those who are most likely to utilise and benefit from them.

In studies with structural data as their primary outcome, there was less consistency in the type of scoring tools used, particularly where efficacy testing of bench models was concerned [5,21,22]. As many of these bench models are newly designed, authors often used customised scales to report on components which were relevant to the model being studied.

The studies with structural data as their primary outcome all concerned the simulation of mitral or other valvular procedures. While there was no one standardised scoring system, commonly assessed structural properties were rupture strength (relating to the chorda tendinae) or elastic modulus (e.g., relating to the leaflets) [21,22] as relating to the real value of these measures in the MV in vivo. Karl et al., 2024 [24] also investigated the ability of their dynamic high-fidelity simulator to mimic real-world resolution of regurgitant fractions and volumes following MV repair.

## 5. Future Directions

The scope of the discussed literature indicates several avenues for future work. It is unclear if confidence levels and self- or senior-evaluated technical scores correlate with the perceived realism of simulators (namely bench models). Such analyses would provide more insight into apt directions for achieving the perfect balance between confidence-inspiring realism and cost, which is often cited as the main barrier to HFS [29,34].

Trainee perception of HFS as superior to other learning strategies due to its novelty may lead to the false deduction that they gain more from HFS than other types of simulation, e.g., LFS, leading them to overestimate their abilities [45]. Therefore, it is essential that levels of perceived realism, confidence level, and skill are interpreted in relation to each other to achieve an ideal simulation which is equally economical and educational [43]. Addressing the barrier of economic transparency is an important step in this.

The reported scales for perceived confidence and skill related to simulation training in minimal access cardiac surgery need to be standardised. One group who may bring this to realisation are the expert workforces of MACS societies, both national and international. Published expert consensus on the appropriate scales for simulation research could have considerable utility in standardising the research in this field for analysis. While simulation platforms are not yet the norm in medical and surgical education, increased interest in simulation training is evidenced by the increase in publications in recent years. Their widespread potential is especially topical given the increasing restrictions and ethical concerns surrounding the use of animal tissue in scientific research [46,47].

## 6. Conclusions

Our review finds a steady increase in publications relating to minimal access cardiac surgery in the past decade, with increasing awareness of novel simulation platforms, including VR and HFS bench models. These models appear to predominantly pursue the simulation of minimal access MV surgery, which may reflect the increasing number of MIMVS cases being performed and a push towards increasing training scope in MIMVS by recent national consensus statements. Technologically innovative platforms are not yet equivalent to wet labs or cadaveric simulation both with regards to perceived utility and conceptual realism. However, using technology as an adjunct may optimise both the time and cost related to delivering gold-standard cadaveric training. With time, increased user familiarity with and economic tailoring of these more novel platforms to surgical need may transform the status quo of how we deliver MICS training: to maximise trainee educational benefit and patient safety.

## Figures and Tables

**Table 1:** The literature on simulation of video-assisted cardiac surgery.

Author	Methods	Simulation Platform	Primary Outcome	Results
Verberkmoes and Verberkmoes-Broeders, 2013 [5]	Low-fidelity simulator for MV and tricuspid surgery N/A participants	MV valvular apparatus created using a baby bottle MV leaflets and orifice created using dental dams	Suture tensions, cost, tactile response, dimensions	Model is very similar to actual anatomical dimensions of the MV Simulation of complete range of Type I and II valve dysfunctions possible (type III simulation limited to IIIa)
Nia et al., 2019 [6]	High-fidelity simulation of MV surgery 99 participants (12 experts, 87 other senior)	Main body consisted of hollow aluminium box and thoracic representation with minimal access port MV, LA, LV, and papillary muscles created from polyurethane foam and cast in silicone Inbuilt software algorithm to differentiate between new and already-placed sutures	Custom Likert scale questionnaire on experience with MV repair and realism of simulator appearance and tactility	Participants agreed that the simulator was a good method for training in minimal access MV surgery The MV and suture placement looked and felt realistic
Jebran et al., 2019 [7]	Low-fidelity simulation of ring annuloplasty, leaflet plasty procedures, neochord implantation, and MV replacement 20 participants (10 residents, 10 students)	Main body aluminium box with fabric dummy/3D-printed alternative representing the valves	Self-reported skill scores and duration of procedures	Members of both groups (residents and students) could significantly improve the time needed for each procedure through the course of the training
Nia et al., 2020 [8]	High-fidelity simulation of MV annuloplasty 102 participants (83 consultants, 12 finished residency, 5 residents)	Model thoracic torso main body with minimal access and robotic access Disposable silicone MV apparatus with a computer-based feedback system	Theoretical pre- and post-assessment, time to place sutures, accuracy of posterior and anterior annular sutures	Participants could work with long-shafted instruments more accurately (suture accuracy 43% vs. 99%) after simulator training
Sobbi et al., 2024 [9]	High-fidelity simulation of MV repair 46 participants (18 experienced, 23 fellows, 5 residents)	3D-printed telesimulator with central chest plate with central opening and surrounding guides for suture placement 3D-printed LV and LA with fittings for disposable mitral and papillary muscles	Anatomical correctness and time taken to place a suture on posterior mitral valve annulus	There was significant improvement in time taken and accuracy to place a suture in the MV annulus after completing simulation training
Sobbi et al., 2024 [10]	3D-printed simulation and telesimulation of MV surgery 11 surgeons	3D-printed telesimulator with central chest plate with central opening and surrounding guides for suture placement 3D-printed LV and LA with fittings for disposable mitral and papillary muscles	5-point Likert scale questionnaire on visual and tactile realism of simulator, suturing experience, overall experience, and confidence pre-and post-telesimulation	There was a significant improvement in the accuracy of annular suture placement and time to place sutures after simulation training
Wang et al., 2024 [11]	High-fidelity simulation of MV repair with patient specific replicas 25 participants (medical students)	Commercially available simulator from Fehling Instruments GmbH & Co. KG, Karlstein, Germany	Custom qualitative and quantitative questionnaires	There was significant improvement in the post-assessment scores of participants who carried out neochorda implantation and annuloplasty sutures The duration of knot tying was shorter after simulation training
En Yean et al., 2024 [12]	Low-fidelity simulation of MV and AV repair and MVR 10 participants (5 trainees and 5 consultants)	Synthetic valve model inside aluminium conduit box	SSES and PCS	The two groups had a significant increase in confidence level in terms of suture placement, ring/valve implantation, and suture knotting using a knot pusher
Azmi et al., 2024 [13]	Low-fidelity beating heart simulation of OPCAB and MICA 2 participants (intermediate trainee and junior consultant)	Modular benchtop simulator using Lego^®^ classic blocks (Billund, Denmark) and Lego^®^ Technic™ parts powered by a Lego Powered UP™ motor and a wireless hub (The LEGO Group) 1″ × 60″ modelling balloons used as conduits and coronaries and immobilised on kitchen sponge	Anastomotic time and score compared with the junior consultant	There were inverse correlations between anastomosis time and number of practices on simulator for both non-beating and beating anastomoses Assessment score increased on average by 26.6%
(Ujihira et al., 2017) [14]	Low-fidelity beating heart simulation of totally endoscopic coronary anastomosis 1 trainee participant performing 100 consecutive coronary anastomoses	Silicone heart in a box Aluminium plate in place of thorax and endoscopic ports	Anastomosis time and number of sutures placed	Significant decrease in average anastomotic time and incidence of vessel injury when comparing first 50 to latter 50 anastomoses by the same trainee Increase in number of stitches placed and average length of coronary incision
Engelhardt et al., 2019 [15]	High-fidelity MIMVS training simulator 12 participants (5 experts and 7 trainees) performed MIMVS	3D-printed MV apparatus using silicon Patient-specific 3D-printed ring prosthesis	Self-reported 5-point Likert scale for usefulness of simulator for different applications, e.g., rehearsal of difficult cases, theoretical knowledge, and practicing with instruments	Trainees were faster at knot tying at the end of simulation training 71% of surgical residents felt better prepared for operating this specific case and associated MV pathology in real-life 100% of experts felt it was an extremely helpful preparation for this specific case

OPCAB, off-pump coronary artery bypass; MICA, minimally invasive coronary anastomosis; MICS, minimally invasive cardiac surgery; MIMVS, minimally invasive mitral valve surgery; LV, left ventricle; LA, left atrium; USB, universal serial bus; 3D, three-dimensional; VATS, video-assisted thoracoscopic surgery; MV, mitral valve.

**Table 2:** The literature on simulation of robot-assisted cardiac surgery.

Author(s)	Methods	Simulation Platform	Primary Outcome	Results
Valdis et al., 2015 [16]	RCT using VR and animal (porcine) simulation 20 participants (trainees) completed IMA harvest and MV annuloplasty	(Heart in a box) porcine chest wall and heart docked onto the da Vinci robot	GEARS	Trainees randomised to the VR group were faster than the control group for both the IMA harvest and MV annuloplasty The VR group scored significantly higher in the intraoperative scoring tool than those who did not undergo VR training
Valdis et al., 2016 [17]	RCT using animal (porcine), wet lab, dry lab, and VR lab 40 participants (trainees) carried out IMA dissection and placed MV annuloplasty sutures	(Heart in a box) porcine chest wall and heart docked onto the da Vinci robot	GEARS	Out of animal (wet lab), dry lab, and VR lab, the trainees randomised to wet lab showed the greatest improvement in TBS and the objective scoring tool compared with the experts The average duration of training was shortest for the dry lab and longest for the VR simulation (1.6 h vs. 9.3 h; *p* < 0.001)
Atroshchenko et al., 2023 [18]	Multicentre trial using animal (porcine) simulation 19 participants (15 trainees and 4 experienced) carried out LA closure, IMA harvest, and MV annular suturing	(Heart in a box) for the LA closure and MV stitches, isolated porcine hearts were placed inside the da Vinci simulator box, and an atriotomy was created to provide access to the MV For IMA harvest, a porcine anterior chest wall with the intact sternum and attached costae in a reversed ‘V’ shape was used	TBS mGEARS	mGEARS could distinguish between novices and experts at left atriotomy and MV annular suturing but not IMA harvesting
Atroshchenko et al., 2025 [19]	Prospective cohort study using animal (porcine) simulation 15 participants (7 cardiac surgeons and 8 non-cardiac surgeons) without prior robotic experience carried out LA closure, IMA harvest, and mitral stitches	(Heart in a box) for the LA closure and MV stitches, isolated porcine hearts were placed inside the da Vinci simulator box, and an atriotomy was created to provide access to the MV For IMA harvest, a porcine anterior chest wall with the intact sternum and attached costae in a reversed ‘V’ shape was used	TBS mGEARS	No significant difference in baseline mean TBS between cardiac and non-cardiac robotic novices 13 out of 15 participants achieved mastery learning level in all 3 tasks on the TBS
Ashraf et al., 2024 [20]	Proof-of-concept study using animal (calf) simulation 50 participants (42 attendings, 8 fellows and residents) carried out IMA harvest	Second- to third-trimester foetal bovine calf thorax model (skin removed) was used. Using straps and lumber obtained at a hardware store, a brace was developed to open the calf thorax while simultaneously securing it to a freestanding table and da Vinci Xi robot	Confidence and realism scales (0–10) before and after simulation	Participants rated the realism of the simulator as 8 out of 10 Participant confidence in robotic IMA harvesting before and after using the simulator increased from a median of 5 to 9 out of 10
Yamada et al., 2017 [21]	Proof-of-concept study using 3D-printed hearts N/A participants MV repair	3D-printed heart and thoracic cavity using CT-derived data and moulded using PVA	Rupture strength Elastic modulus Moisture content	Replica bore tactile similarity to the heart and simulated the difficulty of the surgery as in theatre
Premyodhin et al., 2018 [22]	Proof-of-concept study using low-fidelity simulator 2 participants (fellow and consultant) carried out P2 leaflet foldoplasty and annuloplasty	Polyvinyl alcohol was used to 3D print moulds that were casted with liquid platinum-cure silicone Silicone-moulded MV models were fabricated for 2 morphologies: the normal MV and the P2 flail The moulded valves were plication- and suture-tested in a laparoscopic trainer box with a da Vinci Xi robotic surgical system	Suture feel, tensile strength, and anatomic realism of the models compared to real mitral valve	Evaluators agreed that there was realistic material response to sutures, similarity of tensile strength to MV tissue, and anatomical appearance resembling that of real MVs. Evaluators ‘somewhat agreed’ that the overall model durability was appropriate for training (4.0/5) due to the mounting design.
Al Jamal et al., 2023 [23]	Proof-of-concept study using animal (porcine) simulation 7 participants (various training levels) performed CABG	(Heart in a box) steel skewers used to suspend pig hearts on a Styrofoam board. This is housed inside a cardboard box with openings to mimic minimal access, docked onto a da Vinci Xi robotic surgical system	TECAB questionnaire	Model was easy to set up; anastomotic exercise was realistic and helped to gain confidence.
Karl et al., 2024 [24]	Validity testing of a dynamic high-fidelity simulator 1 participant (cardiac surgeon) performing MIMVS after chorda rupture induced	Dynamic simulator using static setup with metallic holder. In vitro mitral valves 3D-printed according to Engelhardt, 2019	Regurgitant volume (RVol) and fraction (RF) before and after repair using simulator	The RVol and RF decreased to 8 mL and 12% in the repaired valve following simulation. SVP and CO increased to 117 and 4.67, resembling physiological state of valve before chorda rupture was induced

CABG, coronary artery bypass grafting; TECAB, totally endoscopic coronary artery bypass; mGEARS, Modified Global Evaluation Assessment of Robotic Skill; GEARS, Global Evaluation Assessment of Robotic Skill; VR, virtual reality; PVA, polyvinyl alcohol; IMA, internal mammary artery; MV, mitral valve; TBS, time-based scoring; RVol, regurgitant volume; RF, regurgitant fraction; SVP, systolic volume set by the pump; CO, cardiac output.

**Table 3:** Prevalent assessment tools for minimal access cardiac surgery simulation.

Type of Scale	Name	Components
Structural	N/A *	Spatial asymmetry index, tissue trauma events, rupture strength, elastic modulus, moisture content
Psychological	SEES	Variable based on assessed skill. Respondents rank relevant skills on a scale of 1–5, with 1 being not at all confident and 5 being extremely confident
	PCS	Can be paired with technical scores, such as Objective Structured Assessment of Technical Skills, for self-reporting feelings of confidence and competence
	Other	Perception of training Simulation quality
Technical	GEARS	Depth perception, bimanual skill, efficiency, force control, autonomy, robot control
	mGEARS	GEARS with omission of depth perception component
	Other	Anastomotic time, anatomical correctness of sutures

SEES: surgical self-efficacy scale; PCS: perceived competence scale; GEARS: Global Evaluative Assessment of Robotic Skill; mGEARS: modified GEARS. * There is no formal system of assessment for the structural fidelity of surgical simulators as simulator needs vary widely, particularly in MACS. Structural fidelity is tested under the discretion of respective authors as per the type of simulation e.g., elastic modulus in simulation of valve leaflet procedures.

**Table 4:** Studies with inclusion of simulator costs.

Authors	Cost Per Simulator ($)
Verberkmoes and Verberkmoes-Broeders, 2013 [5]	$9
Jebran et al., 2019 [7]	$5000
Azmi et al., 2024 [14]	$179.16
Ashraf et al., 2024 [20]	$133–176
Premyodhin et al., 2018 [22]	$1200 (printer cost) + $2 (disposables)
Al Jamal et al., 2023 [23]	$20
Engelhart et al., 2019 [15]	$3500 (printer cost) + $30–100 (materials)

## Data Availability

No new data were created or analysed in this study. Data sharing is not applicable to this article.

## Supplementary Materials

The following supporting information can be downloaded at: https://www.mdpi.com/article/10.3390/jcdd13050180/s1, Figure S1: Adapted PRISMA diagram showing inclusion and exclusion criteria for article selection during screening process; Table S1: PubMed search strategy <1995 to 1 February 2026>; Table S2: Embase search strategy <1995–2026>; Table S3: Web of Science search strategy <1995–2026>.

## Abbreviations

The following abbreviations are used in this manuscript:

AV	Aortic valve
BISMICS	British and Irish Society for Minimally Invasive Cardiac Surgery
CABG	Coronary artery bypass grafting
CO	Cardiac output
GEARS	Global Evaluative Assessment of Robotic Skill
HFS	High-fidelity simulation
IMA	Internal mammary artery
LFS	Low-fidelity simulation
LVAD	Left-ventricular assist device
MACS	Minimal access cardiac surgery
mGEARS	Modified Global Evaluative Assessment of Robotic Skill
MICA	Minimally invasive coronary anastomosis
MICS	Minimally invasive cardiac surgery
MIDCAB	Minimally invasive direct coronary artery bypass
MIMVS	Minimally invasive mitral valve repair
MV	Mitral valve
MVR	Mitral valve repair
OPCAB	Off-pump coronary artery bypass
OSAT	Objective Structured Assessment of Technical Skills
PCS	Perceived competency scale
PVA	Polyvinyl alcohol
RATS	Robotic-assisted thoracoscopic surgery
RF	Regurgitant fraction
RVOL	Regurgitant volume
RCT	Randomised controlled trial
SSES	Surgical self-efficacy scale
SVP	Stroke volume set by pump
TBS	Time-based score
TECAB	Totally endoscopic coronary artery bypass
TV	Tricuspid valve
VATS	Video-assisted thoracoscopic surgery
VR	Virtual reality
