# Supplementary material for: Simulation Training in Video-Assisted and Robotic-Assisted Cardiac Surgery: A Narrative Review
Source: J Cardiovasc Dev Dis. 2026 Apr 26;13(5):180. doi: 10.3390/jcdd13050180 (PMC13207657; doi:10.3390/jcdd13050180)
Supplement: Supplementary file 1 [file jcdd-13-00180-s001.zip › jcdd-4024432-supplementary.pdf]

Supplementary Materials

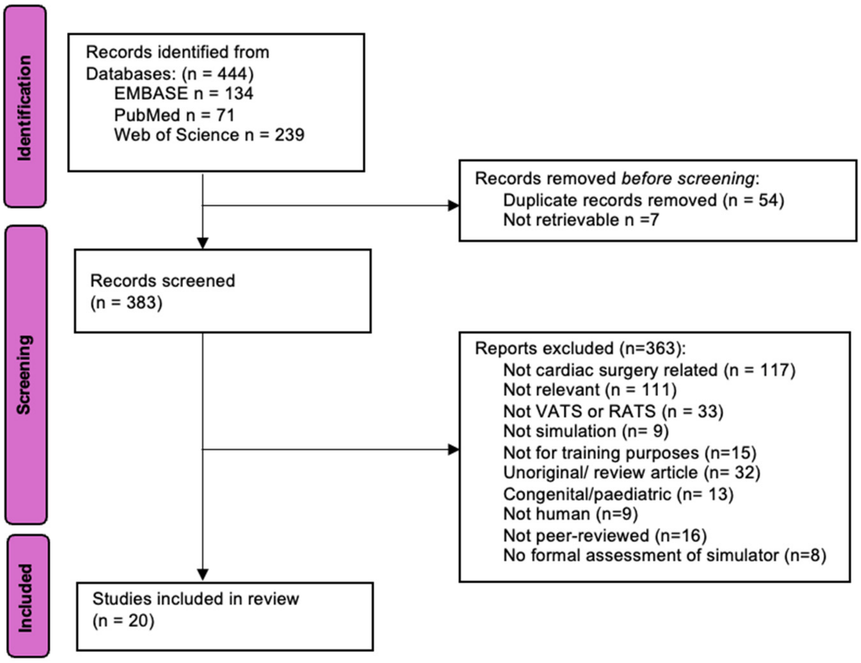

Supplementary Figure S1: Adapted PRISMA diagram showing inclusion and exclusion criteria for article selection during screening process

Supplementary Table S1: PubMed search strategy <1995 to Feb 1, 2026>.

Search	Query	Results
#1	Surgical Procedure, Cardiac OR Surgical Procedures, Cardiac OR Heart Surgical Procedure OR Heart Surgical Procedures OR Procedure, Heart Surgical Procedures, Heart Surgical OR Surgical Procedure, Heart OR Surgical Procedures, Heart OR Cardiac Surgical Procedure OR Procedures, Cardiac Surgical OR Procedure, Cardiac Surgical[MeSH Terms]	302, 994
#2	Patient Simulations OR Simulation, Patient or Simulations Patient[MeSH Terms]	53, 943
#3	Computer Simulations OR Simulation, Computer OR Simulations, Computer OR Computer Models OR Computer Model OR Model, Computer OR In silico Models OR In silico Model OR Model, In silico OR In silico Simulation OR Simulation, In silico OR Computerized Models OR Computerized Model OR Model, Computerized OR Models, Computer OR Computational Modelling OR Modelling, Computational OR In silico Modeling OR Modeling, In silico OR Computational Modeling OR Modeling, Computational[MeSH Terms]	724, 701
#4	Training, Simulation OR Interactive Learning, OR Learning, interactive[MeSH Terms]	55, 702
#5	Minimal Surgical Procedure OR Minimal Surgical Procedures OR Minimally Invasive Surgery OR Minimally Invasive Surgeries OR Surgeries, Minimally Invasive OR Surgery, Minimally Invasive OR Procedure, Minimal Surgical OR Procedures, Minimal Access Surgical OR Procedures, Minimal Surgical OR Procedures, Minimally Invasive Surgical OR Minimally Invasive Surgical Procedure OR Surgical	745, 787

	Procedure, Minimal OR Surgical Procedures, Minimal OR Surgical Procedures, Minimal Access OR Minimal Access Surgical Procedures OR Surgical Procedures, Minimally Invasive[MeSH Terms]	
#6	#1 AND (#2 OR #3 OR #4) AND #5	915
#7	AVR OR Transcatheter Aortic Valve Implantation OR Transcatheter Aortic Valve Replacement[MeSH Terms]	29, 912
#8	TMVR or Transcatheter Mitral valve Implantation OR Transcatheter Mitral Valve Replacement[MeSH Terms]	3990
#9	Laparoscopies OR Peritoneoscopy OR Peritoneoscopies OR Celioscopy OR Celioscopies OR Surgical Procedures, Laparoscopic OR Surgery, Laparoscopic OR Laparoscopic Assisted Surgery OR Laparoscopic Assisted Surgeries OR Surgeries, Laparoscopic Assisted OR Surgery, Laparoscopic Assisted OR Laparoscopic Surgical Procedure OR Laparoscopic Surgery OR Laparoscopic Surgeries OR Surgeries, Laparoscopic OR Procedures, Laparoscopic Surgical OR Surgical Procedure, Laparoscopic OR Laparoscopic Surgical Procedures[MeSH Terms]	188600
#10	#6 NOT (#7 OR #8 #9)	583
#11	#6 NOT (#7 OR #8 #9) Filters: English, Humans	473
#12	Search: Educational Activities OR Activities, Educational OR Activity, Educational OR Educational Activity OR Training Programs OR Programs, Training OR Program, Training OR Training Program OR Workshops OR Workshop[MeSH Terms] Filters: English, Humans	1,569, 209
#13	Search: #18 AND #19 Filters: English, Humans	71

Supplementary Table S2: Embase search strategy <1995-2026>.

1	(Cardiac surgery or heart surgery or cardiac surgical proceures or cardiosurgery or heart operation or myocardial resection or surgery, heart).mp. [mp=title, abstract, heading word, drug trade name, original title, device manufacturer, drug manufacturer, device trade name, keyword heading word, floating subheading word, candidate term word]165374	165374
2	(minimally invasive or (key-hole surgery or keyhole surgery or mini-invasive surgery or mini-invasive surgical approach or mini-invasive surgical procedure or mini-invasive surgical procedures or mini-invasive surgical technique or mini-invasive surgical treatment or miniinvasive surgical intervention or miniinvasive surgical manipulation or miniinvasive surgical method or miniinvasive surgical procedure or miniinvasive surgical technique or miniinvasive surgical treatment or minimal access surgery or minimal access surgical approach or minimal access surgical procedure or minimal access surgical technique or minimal invasion surgery or minimal invasion surgical procedure or minimal invasive operation or minimal invasive surgery or minimal invasive surgical approach or minimal invasive surgical intervention or minimal invasive surgical method or minimal invasive surgical procedure or minimal invasive surgical technique or minimal invasive surgical therapy or minimal invasive surgical treatment or minimally invasive operation or minimally invasive surgical method or minimally invasive surgical methods or minimally invasive surgical procedure or	211886

	minimally invasive surgical procedures or minimally invasive surgical technique or minimally invasive surgical techniques or surgery, minimally invasive or surgical procedures, minimally invasive)).mp. [mp=title, abstract, heading word, drug trade name, original title, device manufacturer, drug manufacturer, device trade name, keyword heading word, floating subheading word, candidate term word]	
3	(simulation or (computational simulation or computer-based simulation or in silico simulation)).mp. [mp=title, abstract, heading word, drug trade name, original title, device manufacturer, drug manufacturer, device trade name, keyword heading word, floating subheading word, candidate term word]	679677
4	(hi-fidelity simulation or high fidelity simulation).mp.	3333
5	(interactive training or simulation-based education or simulation-based learning or simulation-based training).mp.	6444
6	(simulate or simulation model or simulative modeling or simulative modelling).mp.	117733
7	(education using high-fidelity patient simulators or HFPS learning or HFPS training or HFPS-based learning or HFS training or HFS-based course or HFS-based teamwork training or HFS-based training or hi-fidelity manikin-based workshop or hi-fidelity simulation training or high-fidelity manikin-based simulation training or high-fidelity patient simulation learning or high-fidelity patient simulation training or high-fidelity simulation team training or high-fidelity simulation training or high-fidelity simulation-based course or high-fidelity simulation-based curriculum or high-fidelity simulation-based education or high-fidelity simulation-based exercises or high-fidelity simulation-based instructional module or high-fidelity simulation-based learning or high-fidelity simulation-based teaching or high-fidelity simulation-based team training or high-fidelity simulation-based teamwork training or high-fidelity simulation-based training or training using high-fidelity patient simulators).mp.	979
8	(manikin simulation or manikin-based simulation).mp.	134
9	("HFPS (high-fidelity patient simulation)" or hi-fidelity manikin simulation or hi-fidelity manikin-based simulation or high-fidelity manikin simulation or high-fidelity manikin-based simulation).mp.	21
10	(surgery training or surgical education or surgical teaching or training, surgery).mp.	10297
11	1 and 2 and (3 or 4 or 5 or 6 or 7 or 8 or 9 or 10) (Map Term to Subject Heading)	134

Supplementary Table S3: Web of Science search strategy <1995-2026>.

All fields		Cardiac surgery or heart surgery	Search Terms
All fields	And	Minimal access or minimally invasive or robotic-assisted or robotic or RATS or robot assisted or robotic assisted or video assisted or video-assisted or VATS or endoscop* or keyhole or key-hole	-
All fields	And	Simulat*	-
	And	Train*	239
